# A Polymeric Bilayer Multi-Legged Soft Millirobot with Dual Actuation and Humidity Sensing

**DOI:** 10.3390/s21061972

**Published:** 2021-03-11

**Authors:** Shidai Tian, Shijie Li, Yijie Hu, Wei Wang, Aifang Yu, Lingyu Wan, Junyi Zhai

**Affiliations:** 1 Center on Nanoenergy Research, School of Physical Science and Technology, Guangxi University, Nanning 530004, China; tianshidai@binn.cas.cn (S.T.); lishijie@binn.cas.cn (S.L.); huyijie@binn.cas.cn (Y.H.); 2 CAS Center for Excellence in Nanoscience, Beijing Key Laboratory of Micro-nano Energy and Sensor, Beijing Institute of Nanoenergy and Nanosystems, Chinese Academy of Sciences, Beijing 100083, China; weiwang@binn.cas.cn; 3 College of Nanoscience and Technology, University of Chinese Academy of Science, Beijing 100049, China

**Keywords:** soft millirobot, multi-legged, actuator, light-driven, magnetic response

## Abstract

There are numerous works that report wirelessly controlling the locomotion of soft robots through a single actuation method of light or magnetism. However, coupling multiple driving modes to improve the mobility of robots is still in its infancy. Here, we present a soft multi-legged millirobot that can move, climb a slope, swim and detect a signal by near-infrared irradiation (NIR) light or magnetic field dual actuation. Due to the design of the feet structure, our soft millirobot incorporates the advantages of a single actuation mode of light or magnetism. Furthermore, it can execute a compulsory exercise to sense a signal and analyze the ambience fluctuation in a narrow place. This work provides a novel alternative for soft robots to achieve multimode actuation and signal sensing.

## 1. Introduction

The development of artificial intelligence technology has incessantly provoked the soft robotic systems innovation in the past 20 years. Distinct from conventional rigid robots, soft robots have the characteristic of a strong adaptability to external shock. It can complete complex tasks in narrow spaces and unstructured environments, especially in the field of biomedical, human-robot interactions, military and detection [1]. Without a kinematic chain of links, soft robots can perform facial curvilinear motions, including bending [2,3], curling [4,5], twisting [6,7], squirming [2,8,9], etc. Soft robots can adjust their body deformation for adapting to changes in the environment, which paves the way for the establishment of a new type of relationship between the robots and the environment. Generally, a soft robot system is composed of smart materials that can respond to external stimuli such as force, light, pressure, magnetic field, etc. [10,11,12]. It is worth noting that, among these actuation methods, wireless driving technology is favored, because it eliminates the limitation of the working distance and maintains a high degree of freedom of the robot during the execution of the task.

Due to the accurate and rapid modulation by tuning the magnitude, phase and frequency of light, the light-driven mode is commonly used to drive photosensitive robots [3,9,13]. For example, Zuo [6] reported a multi-stimuli-responsive liquid crystal elastomer soft robot system that could modulate deformation by the stimuli of three wavelength bands lights (520, 808 and 980 nm). Their soft robot possessed the capacity to perform a multidirectional movement and shape-morphing modes. Li [14] presented a tri-layered bimorph photothermal robot, including a thermal expansion layer, a passive layer and a cooling layer (carbon black slurry/polyethylene terephthalate (PET)/waterborne acrylic). Under irradiation by the light, their actuator could apply to the photothermal frequency switch, mechanical gripper, soft crawling robot, light-driven mill, etc. The actuation caused by the thermal expansion coefficient mismatch can be found in electrothermal actuators as well [15,16]. To control the robots from a remote distance, with light as the source of stimuli, is undoubtedly the best choice [6]; however, the power of a light-controlled robot is small. Compared to short-wavelength light, near-infrared light stimulation actuators have earned a special position in photothermal devices due to their properties of large bio-penetrability and strong photothermal effect, which is a promising candidate for driving novel micro-biorobots.

The penetrable, fast responsive and nondestructive features of magnetic field determine its superiority to serve a battery-free robot system, especially in a blocked environment. Diverse actuators motivated by a magnetic field have been investigated, which aimed to untether and manipulate soft robots. Hu [7] presented a rectangular untethered small-scale robot that possessed six locomotive modes of motivation, including swimming, walking, climbing, crawling, jumping and rolling. The robot was built from thermo-cured resin (Ecoflex × 10) and neodymium microparticles. Under the magnetic field stimulation, their robot could change locomotion mode between different liquid or solid terrains. Lu [17] developed a millirobot decorated with multiple tapered soft feet architectures to address the challenges of adaptability under wet and dry conditions. As a benefit of the design of the feet structures, their robots could locomote with ultra-fast speed and cross an obstacle easily. Conspicuously, it is a desirable candidate to effectively control magnetically driven robots in a harsh environment. However, as the attenuation of the magnetic field intensity is three orders of magnitude the distance reduction, this modulation is not suited for long-distance control.

Herein, we developed a soft multi-legs robot that can move, climb a slope, swim and sense humidity by near-infrared irradiation (NIR) or magnetic field dual actuation. The robot contains a light-responsive bilayer prepared by a modified polydimethylsiloxane (PDMS) layer and Kapton film and magnetic field responsive multi-legs incorporated with iron microparticles. With the design of the multi-legs structure, our soft robot can decrease the friction during movement in both light and magnetic field stimuli, thus enabling it to move fast and drag heavier objects. The dual-modulation robot combined the advantages between the light-driven mode and magnetic-induced mode, such as long-distance control and moving in a harsh environment. Besides, we also demonstrate that the movement of the robot can sense the humidity in the environment. This work provides a novel alternative for soft robots to achieve multimode actuation and signal sensing.

## 2. Materials and Methods

### 2.1. Materials

Acetylene black (AB, purity >99.9%) and iron powders (99.95% metals basis, density: 7.860 g/cm^3^) were purchased from Alfa Aesar Co. Ltd. (Louis, MO, USA) and Aladdin Co., Ltd. (Shanghai, China), respectively. PDMS (Sylgard 184, Dow Corning Co., Midland, MI, USA) was used as the host matrix for AB particles and iron powders.

### 2.2. Manufacturing Process of the Photothermal Polymeric Bilayer and the Multi-Legged Soft Millirobot

A 25 μm-thick Kapton film was ultrasonically washed with acetone, ethanol and deionized water 20 min successively to remove organic residues and dust on the surface. After drying by air blowing, the Kapton film was fixed onto a glass substrate by using 3-M tapes. The AB particles were dispersed into the PDMS elastomers (2 g) at various concentrations of 1, 2, 3, 4 and 5 wt%. The AB/elastomer mixture was mechanically stirred for 30 min at room temperature; then, the PDMS cross-linker was added with a ratio of 1:10 to the PDMS elastomer and stirred for a further 10 min. After degassed in a vacuum chamber, the AB/PDMS composite was casted on the Kapton/glass substrate and spin-coated. Finally, the AB/PDMS composite was cured at 80 °C for 2 h to fabricate the actuation layer. The polymeric bilayer was peeled off from the glass substrate and cut for further measurements (2 mm × 20 mm). 

**Figure 1:**
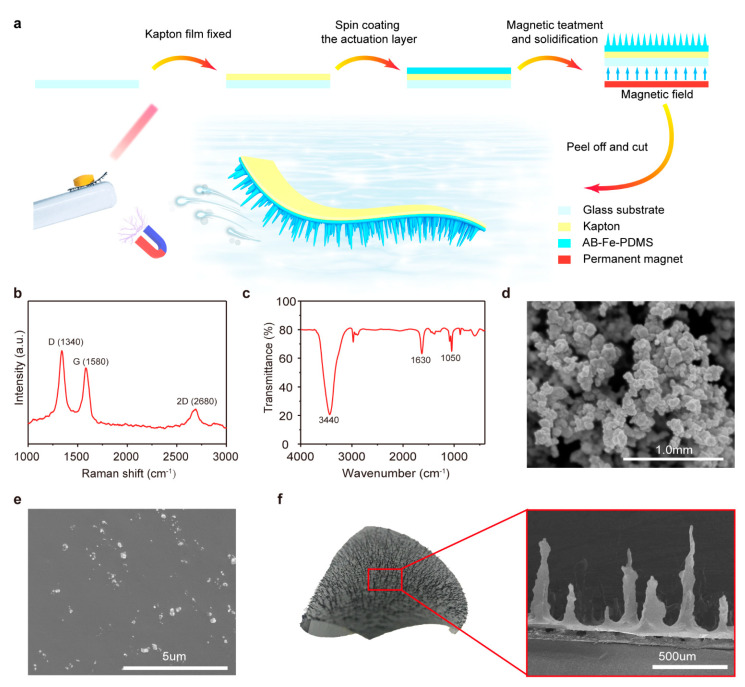
(**a**) Schematic illustration of the synthesis process of the polymeric bilayer actuator and millirobot. (**b**) Raman spectrum of the acetylene black (AB) microparticles. (**c**) Fourier-transform infrared (FTIR) spectrum of AB. (**d**) Scanning electron microscope (SEM) images of acetylene black (AB). (**e**) Cross-section SEM image of the AB/polydimethylsiloxane (PDMS) layer. (**f**) SEM lateral view of the multi-legged structure, showing the irregular tapered architecture of the milli-legs.

To fabricate the polymeric bilayer multi-legged soft robot, 0.5 g iron powder was added to the AB/elastomer mixture. After being stirred, degassed and spin-coated, the same processing mentioned above, a 300 mT commercial permanent magnet was put underneath the substrate with 3 cm distance to induce the formation of quasi-tapered feet into the curing process. Finally, the bilayer with legs was peeled off from the substrate and cut into a rectangular shape (3 mm × 12 mm). Here, a part of the Kapton layer was cut off for convenient observing of the movement of the millirobot under the stimulus of a magnetic field. Figure 1a schematically illustrates the fabrication process of the robot.

### 2.3. NIR Actuation of the Polymeric Bilayer and the Magnetic Field Manipulation

An 808 nm laser (the light spot is around 2 mm × 4 mm) was chosen to control the deflection of the polymeric bilayer actuator. The light density was recorded by an intensity meter (THORLAB PM 200D, Newton, NJ, USA). The real-time temperature mapping of the millirobot upon NIR light irradiation was measured by an infrared camera (Optris PI400, Ferdinand, Germany, test range: −20 °C~100 °C). The highest temperature was obtained by a portable infrared camera (Testo 865, Schwarzwald, Germany, test range: −20 °C~280 °C). The magnetic flux density was measured by a digital Gauss/Tesla meter (Beijing, China), and a linear motor (LinMot E1100, Spreitenbach, Swiss) was employed to control the position of the magnet.

### 2.4. Characterization

Raman spectra was taken by a LabRAM HR Evolution system (HORIBA Co. Ltd., Paris, France) with a 532 nm laser. The Fourier-transform infrared (FTIR) spectroscopy was measured (VERTEX80v, Bruker Co. Ltd., Karlsruhe, Germany). SEM images were characterized by scanning electron microscope (SU-8010, Hitachi Co. Ltd., Tokyo, Japan).

### 2.5. Fabrication of Triboelectric Nanogenerator and Measurements

The triboelectric nanogenerator (TENG) sensing system was fabricated by a simple single-electrode system. Firstly, a piece of copper foil (0.5 cm × 2 cm) was stuck on the ground of an airtight chamber as the electrode of the millirobot TENG. Then, our millirobot was set down on the copper foil and executed exercises under the external stimuli. Lastly, the Cu wire was connected to the copper foil for electrical measurements. A humidifier was put in the chamber to change the relative humidity. The relative humidity in the chamber was measured by a commercial hygrometer. The Current-voltage (I-V) curves were measured by a system electrometer (Keithley 6517, Beaverton, OR, USA).

## 3. Results and Discussion

Carbon-based materials display obvious absorption of the solar broadband spectra [18], Here, acetylene black was employed to fabricate a photothermal actuation device that was principally constructed of two layers: an actuation layer made of AB/PDMS and an inert layer of Kapton film. The coefficient of the thermal expansion (CTE) and Young’s modulus of the AB/PDMS and Kapton layers were different [19,20], which determined the actuator properties under thermal-induced deformation. Attributed to the strong absorption of AB microparticles in the spectra of the NIR region [18], when NIR light was irradiated on the polymeric bilayer actuator, the actuator would experience a fast temperature elevation and then a controllable deformation process.

What is more, the legs played a vital function in the motivation of the millirobot, since it could support the whole weight and, thus, dramatically reduce the friction between the body and the ground. Without the design of the feet structures, the millirobot would be hard to move, even under intense light illumination or a strong magnetic stimulus [17].

### 3.1. Characterization of the Polymeric Bilayer Multi-Legged Soft Robot

Figure 1b shows the Raman spectroscopy of the adopted AB at 532 nm excitation. The AB displayed two well-known strong bands: a G peak around 1580 cm^−1^, which indicates the presence of sp^2^-hybridized carbons, and a D peak around 1350 cm^−1^ ascribed to sp^3^-hybridized carbons [21]. The height ratio between bands D and G (I_D_/I_G_) was around 1.2, indicating that the adopted AB had an excellent order of the graphitic structure [22]. Figure 1c depicts the FTIR spectra of the adopted AB. The peak near 3400 cm^−1^ is attributed to O-H stretching vibration from water absorption or hydroxyl groups present in the AB, while the peaks around 1630 cm^−1^ and 1050 cm^−1^ come from the C=C and C-O stretching vibrations, respectively [21,23,24].

Figure 1d presents the scanning electron microscope (SEM) image of the AB particles. The adopted AB presented a typical assembled spherical structure and a grain size lower than 200 nm. The cross-section SEM image of the AB/PDMS layer (Figure 1e) displayed the AB particles dispersed in the PDMS matrix homogeneously. The feet of the multi-legged soft robot presented a quasi-tapered structure with lengths from 50 μm to 1 mm unequally, as shown in Figure 1f.

### 3.2. Photothermal Actuation of the Polymeric Bilayer under NIR Light

**Figure 2:**
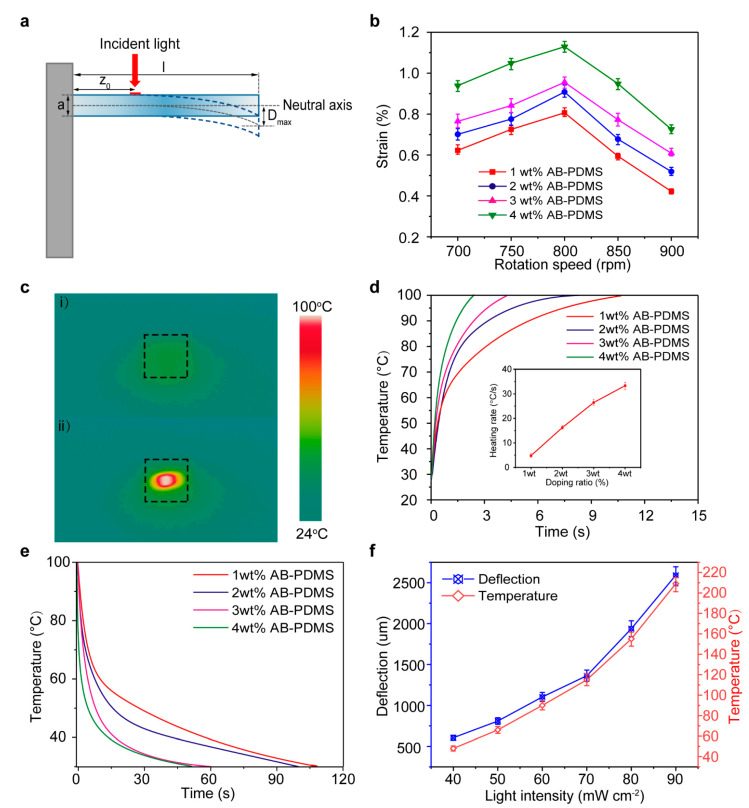
Properties of the bilayer photothermal actuation. (**a**) Schematic drawing for the measurements of the setup. (**b**) Deflection of the bilayer upon the photothermal actuation with different mixing ratios of AB and the rotation speed. (**c**) Thermal image of the bilayer (i) before and (ii) after the infrared irradiation. (**d**) Heating curve of the bilayer with various AB concentrations; the inset shows the heating rate has a linear relationship with the AB composition. (**e**)The cooling curve of the actuator. (**f**) Deflection and the maximum temperature of the bilayer as a function of the near-infrared (NIR) light intensity.

To investigate the photothermal actuation properties of the millirobot, we first examined the properties of the polymeric bilayer without the legs. The length and width of the bilayer were 20 mm and 2 mm, respectively. As shown in Figure 2a, we fixed one side of the bilayer and illuminated the light at selected spots. The photothermal actuation-induced lateral deflection (*D*_max_) at the free edge of the bilayer can be calculated via the Saint-Venant model by [25]:

$$\varepsilon =\mp 3\frac{a}{2l}\frac{D_{\max}}{l}\left(1-\frac{z_{0}}{l}\right)$$

where a and *l* are the thickness and length of the actuator, respectively, *z*_0_ is the distance between the center of the incident light and the fixed edge (*z*_0_ = 5 mm). The negative sign indicates the compressive strain, and the positive sign presents the tensile strain.

Figure 2b depicts the photothermal actuation-induced deflection upon various mixing ratios of AB and the rotation speed under an incident light intensity of 90 mW/cm^2^ irradiation. Apparently, the deflection of the bilayer is proportional to the AB concentration when the concentration is low. However, an irreversible plastic deformation of the polymeric bilayer will emerge once the AB concentration exceeds 5 wt%; the reason for this needs to be further researched. The conversion efficiency of the photo-thermal-mechanical energy of the actuator can be reflected by the strain. The strain was found to be the maximum when the rotation speed was 800 rpm; the thickness ratio of the actuation layer (72 μm) and inert layer (25 μm) was 2.88. In order to investigate the ability of the light-thermal conversion of the bilayer, an infrared camera was employed to record the real-time temperature mapping for the bilayers with different AB concentrations before and after infrared light illumination, as demonstrated in Figure 2c and Movie S1 (Supporting Information). Figure 2d shows the heating curve as a function of the irradiation time from room temperature to 100 °C under the incident NIR light (90 mW/cm^2^). It is obvious that the heating rate increases with the AB concentration increase. The inset of Figure 2d depicts that the heating rate has a linear relationship with the AB concentration, since the increased concentration of AB can convert more infrared phonons into thermal energy [3]. For the adopted AB, under an incident light intensity of 90 mW/cm^2^ irradiation, the maximum temperature of photothermal conversion can reach 210 °C. Besides, the characterization of the cooling process is one of the most important properties of photothermal actuation in practical applications. From Figure 2e, we can conclude that the cooling time decreases with the AB concentration increasing attributed to the excellent thermal conductivity of AB. The real-time temperature curve of the actuator working on the water is displayed in Figure S1 (Supporting Information). It is clearly demonstrated that there is a distinct heat transfer from the actuator to water, and the heat dissipation in the water is much more rapid than in air. To elucidate the effect of the light intensity on the temperature and deflection, we employed NIR with different light intensities ranging from 40 mW/cm^2^ to 90 mW/cm^2^ for the bilayer with a 4 wt% AB concentration (Figure 2f). It can be seen that the light intensity decided the maximum temperature and the deflection of the bilayer. As mentioned above, under the premise of avoiding a bilayer film permanent deformation, the bilayer with 4 wt% AB mass ratios had the best photothermal conversion and light-controlled performance. Thus, the following multi-legged soft millirobot used the same AB concentration to further investigate the locomotion characteristics in the subsequent work.

### 3.3. Fundamental Characteristic of the Polymeric Bilayer Multi-Legged Soft Robot 

**Figure 3:**
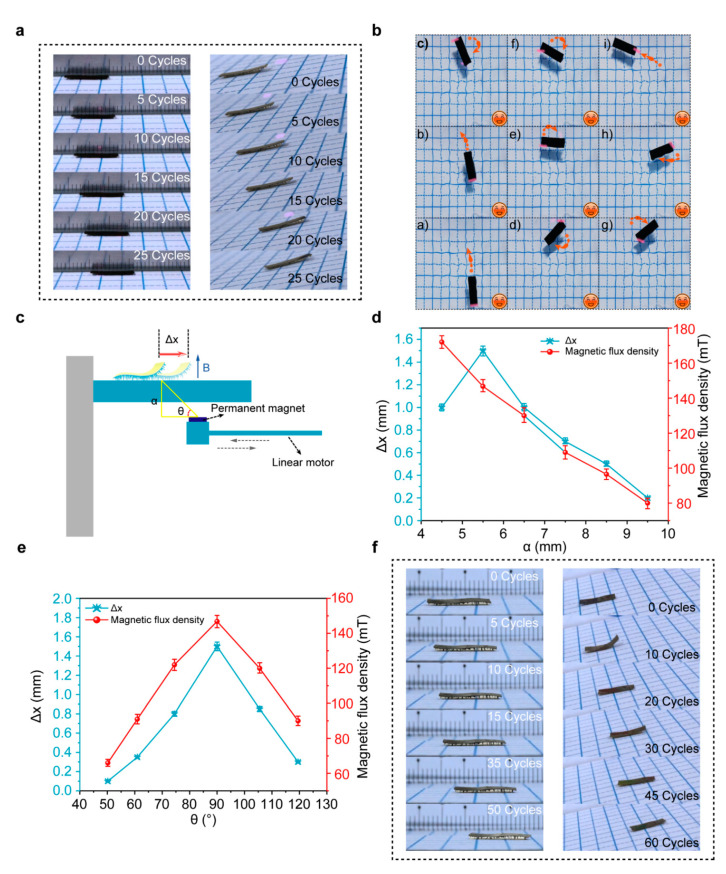
Fundamental characteristic of the multi-legged soft robot. (**a**) The light-driven locomotion of the millirobot moving forward on the plain (**left**) and climbing an 11° rope (**right**). (**b**) Swimming on the surface of the water under NIR light stimulus. (**c**) Schematic drawing for stimulating the soft robot in the magnetic-induced mode. (**d**) Vertical distance (α) between the robot and magnet dependence of the *x*-axis spatial displacement (Δx) of our millirobot in a primary circulation and the corresponding effective magnetic flux density. (**e**) Angle (θ) of the magnetic flux density to provoke the robot determined the *x*-axis spatial displacement and corresponding effective magnetic flux density. (**f**) Magnetically driven motion of the robot walking on a plane (**left**) and climbing a 15° slope (**right**).

A continuous-wave 808-nm NIR light with a light intensity of 90 mW/cm^2^ was employed to investigate the motion properties of the soft millirobot (Figure 3a,b). The laser beam was illuminated at the forebody of the millirobot until the actuation layer achieved sufficient thermal expansion to move forward. When the laser beam was off, the temperature of the millirobot decreased fast and stopped the movement. Such a period of movement of the millirobot was defined as one cycle. From Figure 3a and Supplementary Movie S2, we can see that the robot moved 6 mm after 25 cycles, which was much faster than the squirm locomotion mode reported by Wang [8]. On the other hand, light illumination on the hind millirobot can regulate the millirobot walking in the opposite direction. The capability of climbing slope of our millirobot was also investigated to demonstrate the versatility, as shown in Figure 3a and Supplementary Movie S2. An incline with a slope of 11° was uncomplicatedly climbed by our millirobot, which was comparable to the liquid crystalline elastomer (LCE) soft robot reported by Piotr [9]. Furthermore, a biomimetic micro-fish was designed and modeled, based on the large specific surface area and the hydrophobic properties of the millirobot. As we can see from Figure 3b and Supplementary Movie S2, under the NIR light stimulus, the millirobot can beat water and push itself in the opposite direction. It is a good testimony to the practical applications of our millirobot to swim in a liquid environment.

Apart from the light-driven mode, our millirobot can also be controlled by a magnetic-induced mode; in this case, the magnetic fluctuation can determine the motion state of the millirobot. Analogously, the mobility was investigated to ensure the fundamental capability of the bionics of the millirobot. As shown in Figure 3c, a 300 mT commercial permanent magnet was fixed in a linear motor to motivate the millirobot. Obviously, the magnetic flux density (B) applied to the millirobot fluctuated with the movement of the linear motor back and forth. Generally, the magnetic flux density strengthened when the distance shrank, and the attractive force from the magnet was augmented [26]. The magnetic flux density on the foreleg and the *x*-axis spatial displacement (Δx) was recorded to study the fundamental motion properties of the robot with the primary circulation of the linear motor. An optimal vertical distance (α) between the millirobot and the magnet was investigated in Figure 3d. Distinctly, an excessive attractive force from the magnet hindered the motion of the millirobot, and the optimal vertical distance was ~5 mm. In addition, the angle (θ) of the magnetic flux to provoke the millirobot was explored to obtain the maximum *x*-axis spatial displacement in a motion cycle (Figure 3e). Therefore, the optimum motion mode to motivate our robots was ascertained, with the optimal vertical distance of robot and magnet as ~5 mm and the best actuating angle as 90°. We defined the horizontal direction as the baseline, took 90° as the origin, rotated the direction of the magnetic flux from 90° to 45° and, finally, returned to the original position for a cycle.

In the magnetic field-driven mode, the millirobot can move more controllably and flexibly in comparison to the light-driven mode (Figure 3f and Supplementary Movie S3). In this mode, due to the strong attractive force of the magnet to the millirobot, our millirobot can climb a steeper slope (beyond 15°). Even more, the millirobot can move in the water without considering the effect of hydraulic pressure. A continual fishtail action can be achieved by applying a regular fluctuation magnetic field, which is expected to realize swimming in a liquid environment.

To evaluate the load capacity of the millirobot, we adhered a medical tablet or pill (~60 mg and ~30 mg) on top of the mid-body, which was 20 times and 10 times heavier than its own weight, respectively. The result showed that our millirobot can continually move forward with a cargo load under a light or magnetic field stimulus (as shown in Supplementary Movie S4). This outstanding carrying capacity of the millirobot further proved its potential application in the field of transporting small objects.

### 3.4. Robot Locomotion Analysis and Signal Sensing

**Figure 4:**
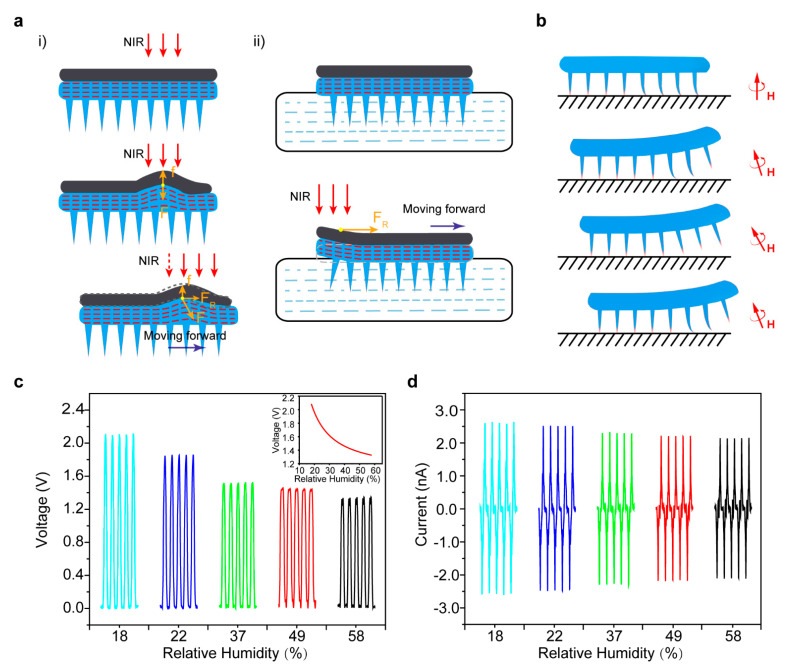
Locomotion analysis of the multi-legged soft millirobot and the signals harvests of millirobot-TENG. (**a**) Schematic diagram of the locomotion mechanism of the light-driven mode (i) walking and (ii) swimming. (**b**) Process of the millirobot motion with the magnetic field fluctuation. (**c**) Voltage signals harvested from the millirobot-TENG in different relative humidities; the inset depicts the fitting curve of the function between the voltage intensity and relative humidity. (**d**) Current signals harvested from millirobot-TENG in different humidities.

In order to understand the locomotion of the millirobots well, we further analyzed the basic mechanism of the robot while moving. For the light-driven mode, when the NIR light illuminated the millirobot, the actuation layer experienced expansion, resulting from the absorbed light of AB. Meanwhile, the inert layer applied a reaction force (F) to balance the swelling force (f) generated by thermal expansion of the actuation layer (Figure 4a(i)). The force balance was broken once the light moved in a forward direction due to the lateral swelling force decreasing with the heat loss. The resultant force (F_R_) would be biased in the same direction. Attributed to the design of the feet structures diminishing the friction against the ground, the resultant force can push the robot forward. In the same way, moving backward can be controlled by moving the light backwards. As for swimming in the water, we inferred that the local force disequilibrium induced by deflection of the millirobot contributed to the swimming capacity of the millirobot [27]. When the NIR light turned on, the edge of the millirobot experienced an upwards movement induced by the photothermal effect (Figure 4a(ii)). The local deflection of the illuminated area caused a forward force and drove the millirobot to move ahead. In addition, only the region stimulated by NIR light was heated, while the temperature change of the water was tiny due to the large heat capacity of water. Therefore, the thermal convection generated from photothermal conversion may slightly affect the millirobot’s swimming ability.

The elastic legs of the millirobot consisted of magnetic particles that deformed and locomoted with the magnetic fluctuation. Initially, the feet of the robot were perpendicular to the magnetic flux and stayed in a static state on the ground. As the permanent magnet turn to 45°, the front feet rose in alinement with the magnetic flux and led the robot forward step by step. After the external magnetic field was back to the original position, the millirobot landed again and prepared the next motion cycle (Figure 4b). In this mode, the millirobot exhibited human-like walking locomotion with the assistance of the feet structures.

It is worth noting that being equipped with an effective sensing system rather than only actuation is an essential requirement for an impeccable robot system [20,28]. Although the sensor technique has quite matured, the limited size for integration and signal transfer still hampers millirobot applications in reality. Considering the characteristics of our multi-legged robot, a millirobot triboelectric nanogenerator (millirobot-TENG) of the single-electrode mode was designed to detect the ambient atmosphere in a narrow space [29]. Our millirobot-TENG can execute compulsory exercise and induce a series of steady triboelectric signal outputs under the stimulation of a magnetic field or NIR light. The schematics of the millirobot-TENG measurement setups can be found in Figure S2 (Supporting Information). Figure 4c,d and Supplementary Movie S5 depict the humidity signals harvested from the millirobot-TENG under the stimulus obtained from executing straightforward head up and down exercises repeated by the millirobot. Ascribed to the structure design of multiple legs for the robot, the contact area between the robot and the electrode can keep an invariableness, thus outputting a series of steady signals during execution of the compulsory exercise. The output voltage intensity of the millirobot-TENG signals decreased from 2.1 V to 1.29 V when the relative humidity (RH) increased from 18% to 58%, which was because the output performance of TENG generally degraded dramatically with the RH increase [30,31]. Interestingly, from the output intensity of our millirobot-TENG, we can conclude the RH value of a narrow space by a contrast analysis of the inset curve of Figure 4c. Combined with triboelectronics, without integrating an extra sensing system, the millirobot can assist the signal harvested to analyze the ambience fluctuations in a narrow space, such as temperature, atmosphere [32,33], etc. Thus, it is conceivable for us to envisage that a robot can load cargo into a narrow place and harvest the ambient fluctuation signals without the integration of complicated sensors.

## 4. Conclusions

In summary, we designed and fabricated a soft multi-legged millirobot that can be motivated by NIR light and a magnetic field. The multi-legged design was introduced to achieve body support and a strong athletic ability of the robot. Under the stimuli, there were several tasks like climbing a slope, fish-like swimming in water and loading a cargo that were realized to demonstrate the versatility of our millirobot. In addition, combined with triboelectronics, our robot can execute compulsory exercise to assist signal sensing without a complicated integration sensor system. This work provided a novel alternative to the soft robot that can achieve multimodal actuation and assist in signal harvesting of the sensing system.

## Supplementary Materials

The following are available online at https://www.mdpi.com/1424-8220/21/6/1972/s1: Figure S1: Temperature change of the actuator working in water, Figure S2: Schematic of the millirobot-TENG measurement setups, Movie S1: Real-time temperature changes of the polymeric bilayer platform with 4 wt% AB concentration under infrared light irradiation (90 mW cm^−2^), Movie S2: Locomotion of the multi-legged millirobot in the light-driven mode, Movie S3: Motion of the multi-legged millirobot in the magnetic-induced mode, Movie S4: Loading capacity test of the multi-legged millirobot, Movie S5: Humidity signals harvested from millirobot-TENG.
