# Supplementary material for: A Polymeric Bilayer Multi-Legged Soft Millirobot with Dual Actuation and Humidity Sensing
Source: Sensors (Basel). 2021 Mar 11;21(6):1972. doi: 10.3390/s21061972 (PMC7998303; doi:10.3390/s21061972)
Supplement: Supplementary file 1 [file sensors-21-01972-s001.pdf]

Supporting Information

A Polymeric Bilayer Multi-Legged Soft Millirobot with Dual Actuation and Humidity Sensing

Shidai Tian [1,2,†], Shijie Li [1,2,†], Yijie Hu [1,2], Wei Wang [2,3], Aifang Yu [1,2,3,*], Lingyu Wan [1,*] and Junyi Zhai [1,2,3,*]

[1] Center on Nanoenergy Research, School of Physical Science and Technology, Guangxi University, Nanning 530004, China; tianshidai@binn.cas.cn (S.T.); lishijie@binn.cas.cn (S.L.); huyijie@binn.cas.cn (Y.H.)

[2] CAS Center for Excellence in Nanoscience, Beijing Key Laboratory of Micro-nano Energy and Sensor, Beijing Institute of Nanoenergy and Nanosystems, Chinese Academy of Sciences, Beijing 100083, China; weiwang@binn.cas.cn

[3] College of Nanoscience and Technology, University of Chinese Academy of Science, Beijing 100049, China

* Correspondence: yuaifang@binn.cas.cn (A.Y.); LYW2017@gxu.edu.cn (L.W.); jyzhai@binn.cas.cn (J.Z.)

† These authors contributed equally to this work.

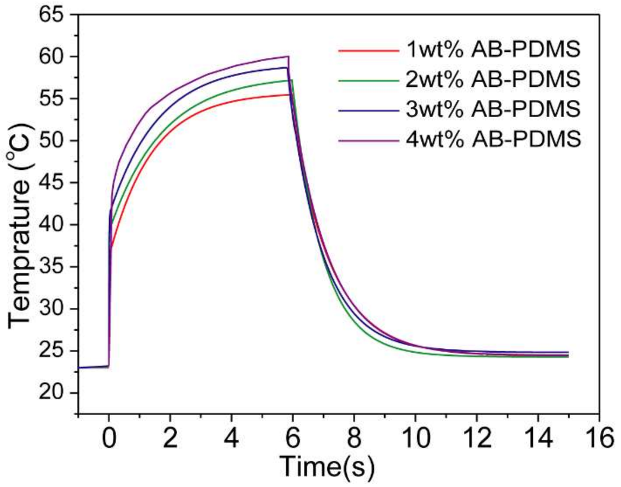

Figure S1. Temperature change of the actuator working in water.

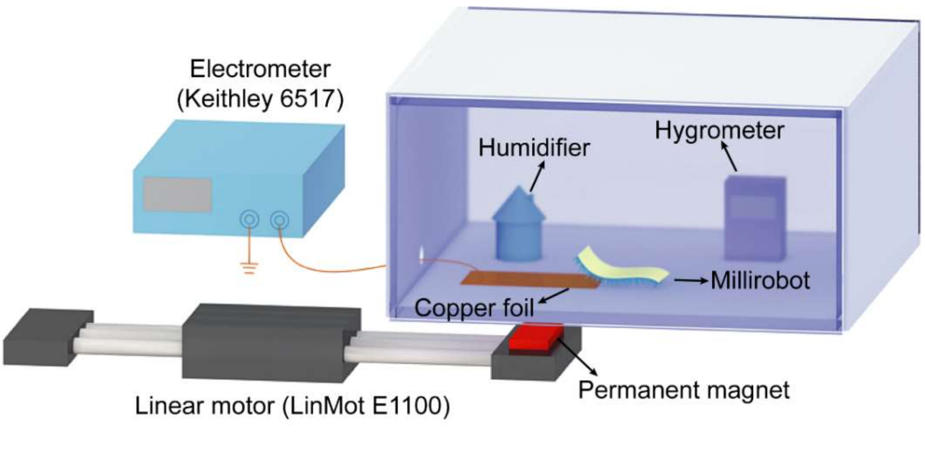

Figure S2. Schematic of the millirobot-TENG measurement set-ups.

sensors MDPI

Supplementary Movies

Movie S1. Real-time temperature changes of the polymeric bilayer platform with 4wt% AB concentration under infrared light irradiation (90mW cm^{-2}).

Movie S2. Locomotion of the multi-legged millirobot in the light-driven mode, including a plane motion, forward, uphill, swimming on the water and turning around.

Movie S3. Motion of the multi-legged millirobot in the magnetic-induced mode, including a plane motion, climbing a slope, walking in the water.

Movie S4. Load capacity test of the multi-legged millirobot, including loading a 30mg pill (10 times heavier of its own weight) in the light-driven mode and loading a 60mg tablet (20 times heavier of its own weight) in the magnetic-induced mode.

Movie S5. Humidity signals harvested from millirobot-TENG under stimulation of a magnetic field and execute the same compulsory exercise in the light-driven mode.
